# Supplementary material for: Breathe with the Waves (BWW)—Creating and Assessing the Potential of a New Stress Management Intervention for Oncology Personnel
Source: Curr Oncol. 2025 Nov 11;32(11):632. doi: 10.3390/curroncol32110632 (PMC12651126; doi:10.3390/curroncol32110632)
Supplement: Supplementary file 1 [file curroncol-32-00632-s001.zip › Supplementary File S1.pdf]

Supplementary File S1: Acceptability, Satisfaction, and Relevance Questionnaire

Table S1. Acceptibility, Satisfaction, and Relevance Questionnaire

Instructions: *Please help us improve our intervention by answering these questions. We are interested in your honest opinions, whether they are positive, negative or neutral. Please answer all of the questions. Thank you very much.*

1. How acceptable do you find this program to be for managing stress?	1 (not at all acceptable)	2	3	4 (moderately acceptable)	5	6	7 (very acceptable)
2. Would this program be appropriate for colleagues who may be experiencing difficulties related to their stress?	1 (not at all appropriate)	2	3	4 (moderately appropriate)	5	6	7 (very appropriate)
3. Do you find this program cruel or unfair?	1 (very cruel or unfair)	2	3	4 (moderately cruel or unfair)	5	6	7 (not at all cruel or unfair)
4. Is this program consistent with common sense or current notions of what a program of this type should be?	1 (not at all coherent)	2	3	4 (moderately coherent)	5	6	7 (very coherent)
5. How humanely does this program treat the participant?	1 (not at all humanely)	2	3	4 (moderately humanely)	5	6	7 (very humanely)
6. To what extent do you think there may be risks in undergoing this program?	1 (very risky)	2	3	4 (moderately risky)	5	6	7 (not at all risky)
7. How much did you appreciate the tools and methods used in this program?	1 (not at all appreciated)	2	3	4 (moderately appreciated)	5	6	7 (very appreciated)
8. Is this program likely to be effective for managing stress?	1 (not at all effective)	2	3	4 (moderately effective)	5	6	7 (very effective)

9. How likely is this program to make lasting improvements for your difficulties related to stress?	1 (not at all likely)	2	3	4 (moderately likely)	5	6	7 (very likely)
10. How likely is it that the program will cause unwanted side effects?	1 (very likely to cause side effects)	2	3	4 (moderately likely to cause side effects)	5	6	7 (not at all likely to cause side effects)
11. How much discomfort is the participant likely to experience while participating in this program?	1 (total discomfort)	2	3	4 (moderate discomfort)	5	6	7 (no discomfort at all)
12. overall, what is your general reaction to this form of program?	1 (very negative)	2	3	4 (indifferent)	5	6	7 (very positive)
**Regarding the program…**							
13. I find the program useful in helping me with my difficulties related to stress.	1 (not at all)	2	3	4	5	6	7 (very much)
14. I would recommend the program to a colleague.	1 (definitely not)	2	3	4 (perhaps)	5	6	7 (definitely)
15. I find the program important for colleagues who work in oncology.	1 (definitely not)	2	3	4 (perhaps)	5	6	7 (definitely)
16. I learned something new from this program.	1 (definitely not)	2	3	4 (perhaps)	5	6	7 (definitely)
17. I feel that this program achieved its goal.	1 (definitely not)	2	3	4 (perhaps)	5	6	7 (definitely)
**Regarding the videos…**							
18. I would watch the program videos or listen to the program audios at home.	1 (definitely not)	2	3	4 (perhaps)	5	6	7 (definitely)

19. I find the videos/audios useful in helping me with my difficulties related to stress.	1 (definitely not)	2	3	4 (perhaps)	5	6	7 (definitely)
20. I would recommend the videos/audios to a colleague.	1 (definitely not)	2	3	4 (perhaps)	5	6	7 (definitely)
21. I find the videos/audios important for colleagues in this situation.	1 (definitely not)	2	3	4 (perhaps)	5	6	7 (definitely)
22. I learned something new from the videos/audios.	1 (definitely not)	2	3	4 (perhaps)	5	6	7 (definitely)
23. I feel that the videos/audios achieved their purpose.	1 (definitely not)	2	3	4 (perhaps)	5	6	7 (definitely)
