# Supplementary material for: Breathe with the Waves (BWW)—Creating and Assessing the Potential of a New Stress Management Intervention for Oncology Personnel
Source: Curr Oncol. 2025 Nov 11;32(11):632. doi: 10.3390/curroncol32110632 (PMC12651126; doi:10.3390/curroncol32110632)
Supplement: Supplementary file 1 [file curroncol-32-00632-s001.zip › Supplementary File S2.pdf]

Supplementary File S2: Sociodemographic Questionnaire

1. Age:
2. Gender:
3. Ethnicity:
4. Job title:
5. Marital status:
6. Which stress-reducing techniques do you currently employ? (mindfulness/meditation, yoga, exercise, breathwork, massage, sauna, cold plunges, other, none)
7. Are you presently receiving any psychological treatment (psychotherapy or medication)?
8. How would you rate your current level of stress on a scale from 0-10? (0 meaning not at all stressed and 10 meaning very severely stressed)
