# Supplementary material for: Breathe with the Waves (BWW)—Creating and Assessing the Potential of a New Stress Management Intervention for Oncology Personnel
Source: Curr Oncol. 2025 Nov 11;32(11):632. doi: 10.3390/curroncol32110632 (PMC12651126; doi:10.3390/curroncol32110632)
Supplement: Supplementary file 1 [file curroncol-32-00632-s001.zip › Supplementary File S3.pdf]

Supplementary File S3: Semi-Structured Interview Guide

1. How do you feel? What is your subjective experience after having completed the breathing exercises?
2. What changes might be associated with this program?
3. What benefits might be associated with this program?
4. What risks or drawbacks might be associated with this program?
5. Do you foresee any other concerns with offering this program in your context? For example, safety or ethical concerns?
6. Is there anything that you would change about the program?
7. If we were to measure the effects of the program in participants, what would you advise us to measure? Any idea is welcome.
8. Might you have any other feedback for us?
