# Supplementary material for: Breathe with the Waves (BWW)—Creating and Assessing the Potential of a New Stress Management Intervention for Oncology Personnel
Source: Curr Oncol. 2025 Nov 11;32(11):632. doi: 10.3390/curroncol32110632 (PMC12651126; doi:10.3390/curroncol32110632)
Supplement: Supplementary file 1 [file curroncol-32-00632-s001.zip › Supplementary File S4.pdf]

Supplementary File S4: Final Synthesized Coding Template

Table S2. Final Synthesized Coding Template.

Theme	Code	Sub-Code	Example Verbatim from the Transcript
1. Benefits	1.1 Stress-reduction benefits		Participant #1: *I admit that out of curiosity, I had my Apple watch which measures my pulse. After each video, I watched it, but really just out of curiosity, and it was consistent with what you were saying in the videos! Like how it* [the exercise taught in the video] *can raise the heart rate a bit, but then drop it back down* [to a lower rate] *again* [after the exercise]. *Well, that could be something to measure* [in future trials].
			Participant #1: *I think it can help me to, yes, really to manage my stress.*
			Participant #4: *It's a kind of fatigue—a good fatigue. How can I put it? A good kind of tiredness. I feel more relaxed.*
			Participant #5: *You know, I can watch that for hours. It really calmed me down a lot.*
			Participant #5: *It's rare—at least for me, in oncology—it's rare to be able to take a moment, and for the hospital to be associated with something calm, something not stressful. So, I think sometimes it might help shift the associations we make with the place we're in.*
			Participant #11: *Blood pressure too, I think — for people who have a bit of an increase in their blood pressure at the start* [before BWW], *but* [with BWW] *breathing we can show that it comes down. I think that would be good, and it's fairly easy to measure.*
			Participant #12: *Clearly a reduction in stress.*
			Participant #14: *It could reduce not only acute, but also chronic stress.*
			Participant #14: *I think it can improve the quality of life for people who feel really stressed.*
			Participant #15: *Right now, I feel really Zen. Yeah, I had a shift. I was feeling pretty stressed all day today. And now, it feels really good to feel, like, really Zen.*
			Participant #18: *Stress management, like at work. I think that it could be a great tool.*
			Participant #20: *It really works. It relaxes you. It's incredible. I feel so relaxed right now.*
			Participant #26: *I feel good, relaxed, centered. I almost fell asleep with that. It really works.*
			Participant #28a: *Oh, I'm sure it's going to calm my nerves. Definitely.*
			Participant #29: *I feel lighter, I can feel it. It's like there's a feeling… like a wave passed through and took away the things that were bothering my mind. And it's like I came back to the present moment more easily than before the exercises. I feel like I'm already more grounded in my movements and my voice. It's as if a sense of calm has settled in. So, it's a feeling of well-being and comfort—cozy, gentle, restful. It's like a slowness has taken hold of*

	me, inhabits me, and makes me speak more slowly than usual. It's… it's… it's really a pleasant sensation.
1.2 Work-performance benefits	Participant #1: [BWW could lead to] *even better efficiency at work. I think it* [the new program] *can, you know, when you just can't manage to write your reports or when you know that you've had one meeting after another with your patients* […] *You know, in my case, as a social worker, well that's what I do, I meet the parents* [of children with cancer]. *I can have several meetings in a day. Between two meetings you can practice the new exercises to become again ... how can I say ... a better, we always say that we are 'our own tool', so to become a better tool.*
	Participant #11: *Well, I think that when we're really stressed, we might react poorly, misinterpret situations, take things more personally—even when they're not meant that way—because we work in an environment where everyone is stressed, whether it's other colleagues or especially the families and patients. So, I think if everyone were more calm, it would calm the patients, it would calm the families. […] Everyone would feel better.*
	Participant #12: *The preparation for a situation that might be difficult. The reaction to a situation that might be difficult—these are all elements that can, I think, be made easier by these practices.*
	Participant #13: *Well, of course, if we're more relaxed, we're better able to provide care, which means better patient care in the end, and better support for our colleagues.*
	Participant #13: [BWW can be used to] *help calm things down and be more relaxed to carry out the next steps of our care.*
	Participant #18: *It can be good just to calm your mind before starting to do something again and to prepare yourself.*
1.3 Mindfulness benefits	Participant #12: [BWW catalyzes] *a better connection with your feelings, on the mindfulness aspect.* [...] *To take a break to try to observe yourself, to listen to the voices in your head. Mindfulness can be facilitated by this kind of exercise.*
	Participant #24: *The sensations of being here and now, of being more fully present with our clients.*
	Participant #27: *It's like when we breathe, we become aware or mindful of our physical tension.*
	Participant #29: *I feel like I've returned, somehow, to the present moment, more easily than before the exercises. It's like I'm already more grounded in my gestures and my voice.* […] I*t's like there's a slowness that has also settled in me, that inhabits me and makes me speak more slowly than usual. It's... it's... it's really a pleasant sensation.*

			Participant #30: *For me personally, I think I already had a bit of a foundation when it comes to breathing, meditation, and all that, but it actually reminds me of a lot of important things.*
			Participant #30: *Well, I think that in general, it's pretty easy—in the routine, in the stress, in the different situations we deal with in oncology—to just stay in our heads ... and kind of just endure the stress. Whereas we can actually take a bit more control—not necessarily by welcoming the stress, but by being more aware of how our body is experiencing it, and by having an impact on it.*
2. Challenges	2.1 Challenges experienced	2.1.1 Acknowledging Stress	Participant #1: *It's just that, in the moment, personally, it made me confront the fact that I was really noticing how shallow my breathing was. And then it's like… ugh… it's like you're coming face to face with your stress*
		2.1.2 Dizzy	Participant #4: *After my first video, I felt dizzy.*
			Participant #20: *But it's true that during the first one, if the pace is too fast, it can... I didn't get dizzy, but I was close, and my heart was beating faster—even though I was going at my own pace.*
			Participant #27: *I felt a bit dizzy. So, you know, if it's not done the right way or if it's done too quickly, uh, it can cause discomfort, maybe especially for certain people.*
			Participant #28b: *And maybe sometimes, depending on the exercises, it can make you feel a bit lightheaded.*
		2.1.3 Tired	Participant #13: *I don't see any immediate risks, except maybe wanting to have a bed right afterward to sleep.* [Laughs].
			Participant #26: *I almost fell asleep*
			Participant #28b: *I feel like each exercise had a different effect. For example, after the first one, I felt more calm but still with some energy. Yes. After the second, I just felt calm, and a bit sleepy after the third.*
			Participant #28b: *I feel like it kind of drained me.*
	2.2 Anticipated challenges	2.2.1 Potential Danger	Participant #3: *Maybe for some people who aren't used to it … they might actually lose consciousness because there's too much oxygen.*
			Participant #4: *Maybe the only point to look at—like, are people really aware of what's going on in their own body?*
		2.2.2 Resistance Based on Personality Type	Participant #12: *From time to time I get responses* [when suggesting yoga] *like, "Yes, but that really doesn't work for me." There are people who just can't… I don't know if… maybe they're people who always need to be in action and have a hard time taking a moment to pause. But I've already seen several people who tell me, "Yeah, no, that's really not for me." So, for me, it's not that it would be a potential* risk, *but I see it more as a potential barrier or*

			limitation — something that might not be applicable to everyone. Uh, yes, some people seem a little more, um, resistant.
3. Suggested changes	3.1 Skip Intro		Participant #1: *I don't know if there could be a way to skip the first minute of each video once we've already heard it. Yeah. Otherwise, we can always just move the cursor to where it needs to be.*
			Participant #28b: *Just if it's the same videos I'm redoing, just the intro part. Maybe if I want to do it quickly—for example, in the bathroom—it'd be good to know where to skip to once the intro is over so I can go straight to it.*
	3.2 Advise Not to Practice in Dangerous Contexts		Participant #14: *It's just that breathing exercises, obviously, shouldn't be done while driving, for example, especially if you're closing your eyes.*
	3.3 Desire for more or less guidance		Participant #20: *It's true that you don't give any instructions in the first one. Okay, now I ... Okay, I understand why. Because someone like me, who doesn't have much experience, really appreciates having a guide.*
			Participant #28b: *I just think it's nice that you did it with us. Because I'm kind of shy, and sometimes I felt a bit self-conscious, and hearing that you were doing it too reassured me. I thought, "Okay, I'm not the only one."*
